# Catalyzing Technology-Based Innovation in Teen Pregnancy Prevention: an Implementation Model and Findings from a Human-Centered Design Initiative

**DOI:** 10.1007/s11121-023-01523-3

**Published:** 2023-07-19

**Authors:** Jill Antonishak, Katy Suellentrop, Riley J. Steiner, Laura Lloyd, Sarah M. Axelson

**Affiliations:** 1 Three Flights, LLC, Washington, DC USA; 2 RTI International, Research Triangle Park, NC USA; 3 Power to Decide, Washington, DC USA; 4 Independent Consultant, Washington, USA

**Keywords:** Teen Pregnancy Prevention, Interventions, Human-centered design, Innovation

## Abstract

From 2015 to 2021, the US Department of Health and Human Services’ Teen Pregnancy Prevention (TPP) program funded Power to Decide, a national reproductive health nonprofit organization, to catalyze innovation in adolescent sexual and reproductive health through the development of technology-based interventions. Power to Decide’s initiative, Innovation Next, supported twenty innovation teams in using human-centered design (HCD) to develop new products, services, and programs. We describe the Innovation Next implementation model, which can inform future efforts to develop innovative, technology-based TPP programs using HCD. To that end, we draw on quantitative and qualitative data collected for program improvement to summarize key implementation findings.

## Introduction

The national Teen Pregnancy Prevention (TPP) program directs federal funding to support the implementation of programs with evidence of effectiveness. Since its inception in 2010, the TPP program has served more than 1.4 million young people across the USA (Office of Population Affairs, 2022), and the birth rate among 15- to 19-year-olds has continued to decline dramatically during this time (Osterman et al., 2022). Despite the number of evidence-based TPP programs available, there remains a need for innovation in TPP programming to ensure effectiveness across various implementation settings and adolescent populations as well as ongoing relevance.

Given the widespread use of technology among adolescents, including for health information-seeking, technology-based interventions represent an important opportunity for innovation in TPP programs. Most adolescents have access to technological devices, regardless of socioeconomic status (Anderson & Jiang, 2018), so implementing programs digitally has the potential to reach large numbers of young people and address gaps for adolescents who may not have access to effective, in-person TPP programs. Moreover, there is growing evidence that technology-based sexual and reproductive health interventions can positively change attitudes, behavior, and health outcomes, including reducing unintended pregnancies (Antonishak et al., 2015; Widman et al., 2018).

Beginning in 2015, the US Department of Health and Human Services’ TPP program funded Power to Decide, a national reproductive health nonprofit organization, to catalyze innovation in adolescent sexual and reproductive health through the development of technology-based interventions. To do so, Power to Decide developed Innovation Next — an incubator program that teaches and supports innovation teams to use human-centered design (HCD) to develop new products, services, and programs. HCD, also referred to as design thinking, is an intentional process that supports innovation, and it has increasingly been used to develop social service and public health programs (Abookire et al., 2020; Brown, 2008; Brown & Wyatt, 2010; Nandan et al., 2020; Razzouk & Shute, 2012), including effective sexual and reproductive health interventions (Antonishak et al., 2015; Bazzano et al., 2017). This paper describes the Innovation Next implementation model and findings to inform future efforts to develop innovative technology-based, TPP programs using HCD.

## Implementation Model

**Fig. 1:**
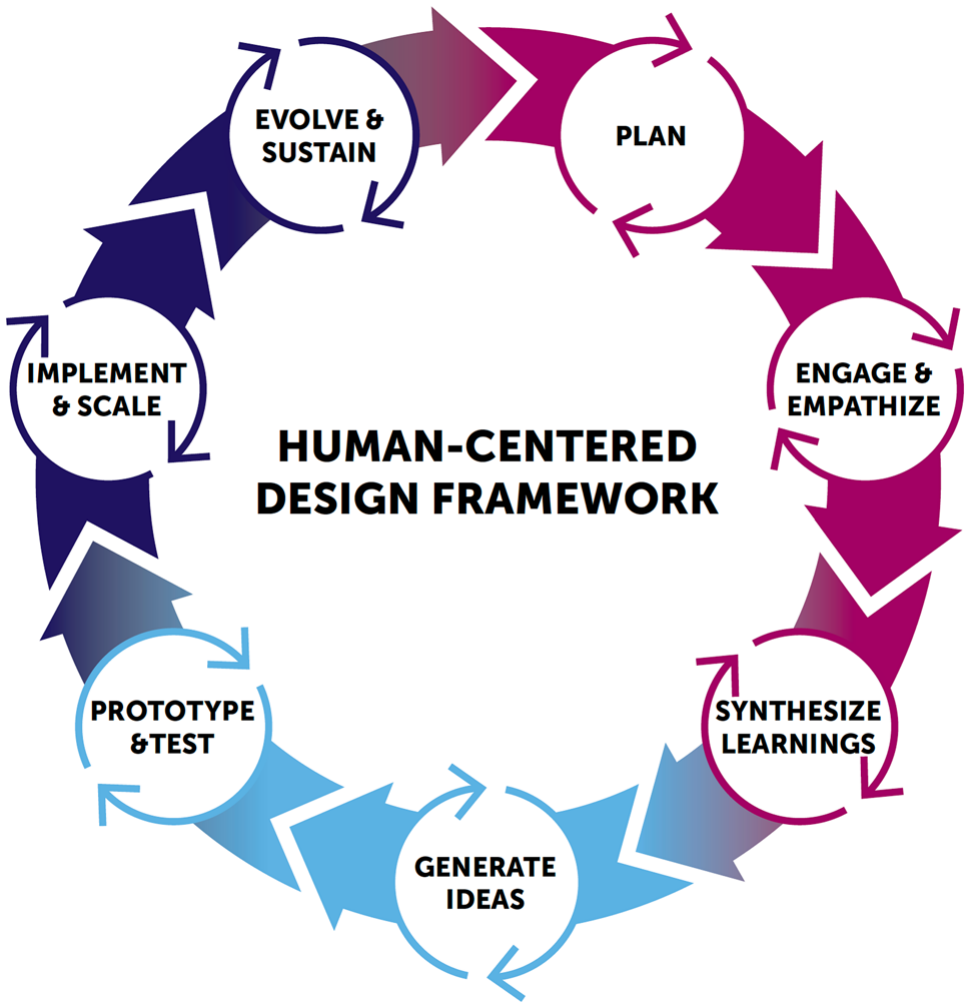
Power to Decide’s human-centered design framework

Innovation Next is a unique incubator program that provides HCD training and customized coaching to multidisciplinary teams as they develop technology-based interventions to prevent teen pregnancy. HCD originated as an approach used in business and technology to support the development of innovative solutions that address the needs of end users (i.e., people who will “use” the solution). Power to Decide developed an adapted model of HCD for Innovation Next that emphasizes the iterative nature of the design process (Fig. 1). In this approach, innovators develop a plan by clearly identifying a design challenge and assembling a multidisciplinary team. They engage with end users as deeply and as often as possible in order to gain radical empathy, an essential component of HCD. Radical empathy involves recognizing and understanding one’s own biases and gaining deep awareness and understanding of the perspectives and experiences of others (Lubana, 2021). The goal of gaining radical empathy is to use that understanding to design meaningful products and services that meet end users’ needs, rather than assuming a solution developed by program designers/developers, who may not have relevant lived experience, will address the need. Synthesizing and applying the learnings from end user engagement, the team generates as many ideas as possible on how to address the identified challenge. In this stage, iteration is a critical tool, as innovators select the ideas that seem the most promising, turn them into low-fidelity prototypes (i.e., simple, inexpensive, limited-function versions of a product or program), test them with end users, and refine them based on the feedback they receive. Embracing the ambiguity of a variety of potential solutions during this process allows innovators to iteratively narrow in on one idea or solution that is most likely to effectively meet end users’ needs. The concept is finalized, implemented, and scaled over time. Importantly, innovators continue to monitor changes in end-users’ needs and desires over time to support adaptation of the program, product, or service as needed. Recent applications of HCD suggest that this process may offer a more resource-efficient way of developing public health interventions and yield higher end user satisfaction as compared to more traditional development methods (e.g., theory-based curriculum design) (Abookire et al., 2020).

Drawing on previous success using HCD to support sexual and reproductive health (Antonishak et al., 2015), Power to Decide established the Innovation Next program to catalyze technology-based innovation in TPP programs by recruiting, training, and supporting innovators to apply the framework outlined above. The program began with a call for online, written applications from three-person, multidisciplinary teams of innovators. All team members had to be located in and focused on preventing teen pregnancy in the US. They were also required to articulate a particular challenge within the teen pregnancy prevention field, framed as a “How might we…” question, for which they would work to develop a solution (see Table 1 for the questions posed by selected teams and resulting emerging innovations). Teams then completed a video interview with a panel of HCD experts to discuss their applications in more detail. The application process was intentionally structured to assess openness to the use of HCD, diversity of skill sets across the team, diversity of challenges teams proposed to address, end user audience across the applicant pool, and potential for impact. Selected teams included innovators from nonprofit organizations, state and local agencies (e.g., health departments), academic institutions, and private for-profit organizations (or some mixture thereof). In total, 20 teams participated in Innovation Next across 5 years — Cohort 1 began with 10 teams and was narrowed down to five (additional details below), while Cohorts 2 and 3 each had five teams.

**Table 1:** Challenges identified by teams selected to participate in the Innovation Next incubator and resulting emerging innovations

**Cohort**	**“How might we” Question (from application)**	**Emerging Innovation**
Cohort 1	How might we (HMW) catalyze parents, guardians, and other supportive adults to promote positive sexual health among young people and reduce teen pregnancy?	(Not selected to move to Stage 2)
	HMW connect with teens who can’t, won’t, or simply don’t seek contraceptive services through available resources, clinics, and schools?	BC4U — A scheduling system that allows teens to book appointments in an easy and confidential fashion; key design components include not requiring a log in which reduces barriers to entry, a chat function, the ability to schedule a visit with a friend, and information about the clinicians that are available
	HMW reduce pregnancies and STIs in youth in, or formerly in, foster care by helping them articulate, set, and adhere to personal rules for healthy romantic relationships?	(Not selected to move to Stage 2)
	HMW better enable pediatricians and primary care providers to talk to their adolescent patients about sensitive matters of sexuality and pregnancy prevention, identify needs and preferences, prescribe contraception if needed, and supply appropriate resources?	Starting the Conversation — A software application package that includes an electronic pre-visit psychosocial assessment for adolescent patients that is connected to an EHR-integrated clinical decision support system for pediatric providers
	HMW create a sex education resource that is as engaging as Facebook?	okayso — An app that allows young people to ask questions about sexual health, pregnancy prevention, relationships, and more, and for experts to reply with tailored responses
	HMW use technology to create a program that individualizes sex education for different groups of students in order to reduce disparities in sexual health outcomes?	Real Talk — A mobile app that connects teens 13–15 years old with authentic, crowdsourced stories and trusted resources on sensitive topics like puberty, mental health, bullying, and healthy relationships, to help them know they are not alone
	HMW engage Latino tweens and teens, along with important people in their social networks, to co-create a sustainable platform to share compelling stories, build critical skills, and take real actions to promote their sexual and reproductive health?	(Not selected to move to Stage 2)
	HMW reduce the rate of teen pregnancy by providing teens with the knowledge and supplies to lead healthy, sex-positive lives?	(Not selected to move to Stage 2)
	HMW leverage digital media to give Hispanic/Latino parents the skills and tools needed to effectively talk to their kids about pregnancy prevention?	Momentos — A Spanish-language text messaging service that provides weekly, practical advice to parents; texts are customizable based on the age and gender of the children, and guide parents through teaching about sexual health and preventing unplanned pregnancies
	HMW catalyze parents, guardians, and other supportive adults to promote positive sexual health among young people and reduce teen pregnancy?	(Not selected to move to Stage 2)
Cohort 2	HMW better increase accessibility to emergency contraception for low-income teens in urban areas?	Miss Morning After — A digital toolkit for pharmacists to increase their ability to be teen-friendly and offer the support services that teens need when navigating access to emergency contraception; the youth-friendly pharmacy toolkit includes shelf talkers, informational brochures, stickers, staff training materials, and staff break-room posters
	HMW bring medically accurate, culturally appropriate, cost-effective, and realistically sustainable adolescent sexual health information to the rural and underserved youth of the Navajo Nation?	Dine Youth Network — An engaging, first-of-its-kind interactive website with games, exciting videos about Navajo culture, and current medical information, designed specifically by and for Navajo Nation Youth
	HMW use technology to create a holistic health and wellness program to support and empower teen parents?	Young Parents United — A website and app that allow young parents aged 16 to 21 overcome isolation and stigma by providing accurate information and connection with peers
	HMW increase teens’ sense of agency and efficacy over their sexual/reproductive health and mental health, thus reducing STIs and teen pregnancy in under-served communities of color?	The Mind Elevation (ME.) Project — Six virtual “characters” from rural Mississippi who deal with a range of issues such as consent, relationships, body image, pregnancy prevention, and abuse; the ME. Project shares stories on the social media channels that youth frequent, such as Facebook, Snapchat, Instagram, and YouTube, as a means to link youth to vetted, high-quality, information and resources on mental health and pregnancy prevention
	HMW enable parents to customize their child's sexual health curriculum to meet family needs and aspirations?	Bloom — A three-part curriculum for youth ages 5–10, 7–12, and 9–14 and their parents, designed to help families have conversations about sexuality; each playbook is filled with relevant, age-appropriate activities and prompts for parents and grade school-aged kids to work on together, focused on the topics of biology, safety, and empathy
Cohort 3	HMW create culturally and linguistically relevant opportunities and materials for young refugee women aged 18–24 in the USA?	Hello, America — A YouTube channel that uses video storytelling, teeming with diverse stories from the lives of refugee women in America, told through their voices; the series broadly explores life in America through a variety of topics like activities, school, sports, beauty, food, and more, and through those ‘general interest’ segments (beauty, cooking, etc.), delivers teen pregnancy prevention information, education, and messaging in subtle “culturally appropriate” ways
	HMW use digital media to thoughtfully engage adolescents in experiences of teenage pregnancy?	Layla’s Last Week — An interactive webcomic where the user guides the story of a 17-year-old named Layla, juggling her job, school, family, and a first partner, designed to allow users to safely practice vital pregnancy prevention skills in a relatable form of entertainment media
	HMW employ digital learning tools to expand and enrich access to sexual and reproductive health education for adolescents and young adults with diverse learning needs?	Teen Table Talk — A sex-ed story-sharing platform utilizing peer mentorship and Universal Design for Learning (UDL) to provide pregnancy prevention information in an accessible way to diverse learners
	HMW leverage existing entertainment preferences to engage young men in sexual health education?	Wingman — A smartphone keyboard that young men can deploy into their common texting experiences to help facilitate conversations about pregnancy prevention, STIs, contraception, and consent; at any point during a text exchange, Wingman can be deployed from the mobile phone’s keyboard interface to offer users multiple content options and tools to make texting fun, while promoting open and clear communication about the topics noted above
	HMW increase dual method use and subsequently decrease teen pregnancy among 15–18-year-old Black and Latinx adolescents?	Oyeah — An immersive video game that addresses the pain points heard from teens, including a desire to conduct risk assessments with their parents but a lack of understanding of how to do so accurately; uncertainty and discomfort communicating with partners about dual method use and STIs, and “separate worlds” in which users assume their partner is doing what they need to do about pregnancy prevention and the user only needs to worry about themselves and their own actions

**Fig. 2:**
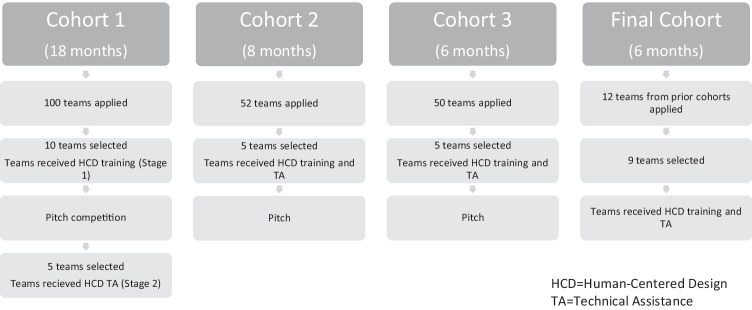
Innovation Next implementation

Once teams were selected, they participated in the Innovation Next incubator. Embracing the HCD principle of iteration, the design and length of the incubator was tested and refined over the three cohorts to develop the final implementation model (Fig. 2). For Cohort 1, the incubator lasted 18 months and used a multi-stage process, including HCD training (Stage 1), a pitch competition, and customized HCD technical assistance (TA) (Stage 2). During Stage 1, teams attended two intensive in-person workshops on the HCD process facilitated by Power to Decide and IDEO, a global design firm that developed the original mouse for Apple computers and has a continued a legacy of high-quality, user-centered products and experiences. The first workshop focused primarily on HCD activities related to planning through generating ideas (Fig. 1). Participants were introduced to HCD tools and strategies and provided opportunities for guided practice and feedback from dedicated “innovation coaches” — Power to Decide staff with expertise in HCD. For example, teams conducted mock end user interviews to practice skills and receive feedback from coaches. They also learned about HCD-specific strategies to gain empathy, such as journey mapping, which involves developing a visual representation of the process that an end user goes through to reach a goal or complete a task, and analogous experiences, which involve identifying and participating in an activity in an analogous context that might evoke similar emotions to those of the end user in order to provoke new understanding and thinking. For example, one team explored trapeze classes as an analogous experience for using a condom for the first time; the classes carried risk and involved “unfamiliar equipment” that they had to learn how to use during a potentially high-stress time. Teams also built and tested low-fidelity prototypes, using feedback tools developed by Power to Decide, to briefly introduce them to the prototyping and testing activities that would be the focus of the second workshop. Such activities were designed to give teams the skills and confidence to apply the HCD strategies they learned during the workshops on their own. The second workshop focused primarily on HCD activities related to synthesizing learnings through prototyping and testing (Fig. 1). Teams learned how to synthesize a variety of learnings from numerous end user engagements of different lengths, frequencies, and depths (e.g., end user interviews, journey mapping, observations, analogous experiences, group interviews, etc.) Teams also learned how to iteratively develop higher-fidelity prototypes in order to move toward a solution. Between the workshops, teams received TA from their innovation coaches. Most often delivered in the form of weekly coaching calls, the TA was guided by the unique needs of each team as they applied HCD tools and strategies to their selected challenge. Common discussions included reflecting on learnings from end user engagements, strategizing for future end user engagement activities, and creative problem solving. Following Stage 1 (which lasted 6 months), each of the ten teams developed a business plan and delivered a “Shark Tank” style oral pitch during which they shared the findings from their end user research and their resulting innovation concept. The business plan supported the pitch and provided additional details about how the innovation would move from concept to minimum viable product (MVP), referring to a new product with sufficient features to be functional for end users’ engagement and inform future iterations. From the competitive pitch, the five highest scoring teams moved to Stage 2 (12 months), during which they received additional funding and TA (e.g., coaching calls, webinars).

For Cohort 2, the implementation model was adapted to include fewer teams and a shorter time frame, and to eliminate the distinct stages and competition between teams. Rather, all five teams selected from the application process attended the two intensive in-person workshops (over 8 months). Building internal capacity and expertise in HCD, Power to Decide took the lead on workshop design and delivery with limited support from IDEO, and the workshops were adjusted to provide even more hands-on experience with HCD. For example, rather than conducting mock end user interviews with Power to Decide staff, teams conducted end user interviews with young people and received feedback from coaches who observed the interviews. Additionally, workshop participants took part in an “immersive experience” (i.e., experiencing a similar program or service first-hand, a technique frequently used in HCD), in which all participants went to an adult sex education workshop together. The goal was to reflect on the feelings such an experience elicited as adults (e.g., awkwardness, discomfort), how those feelings might be amplified for youth receiving sex education, and how that might influence intervention design. Between the workshops, teams received tailored TA from the Power to Decide innovation coaches. Given that HCD is an extremely flexible and adaptive approach, coaching had to follow suit in order to be responsive to teams’ needs for support. The pitch was moved to the end of the incubator program to highlight and share the concepts that teams had developed. This revised model was also used for Cohort 3, which was implemented over a slightly condensed time frame (6 months), and the workshops were designed and delivered entirely by Power to Decide.

Finally, during the last year of the project, all teams from each of the first three cohorts had the opportunity to apply for additional funding to bring their innovation to market or scale. The teams selected for additional funding included two teams from Cohort 1, four teams from Cohort 2, and three teams from Cohort 3. During this final cohort (6 months), they received training and TA from Power to Decide and staff from a new nonprofit partner organization, SEED SPOT, which supports entrepreneurs creating solutions to social problems. This TA included weekly and monthly coaching calls, webinars, and informal “coffee chats,” in which teams could identify and discuss topics relevant to their cohort.

Innovation Next required a significant investment of time and resources. The federal award provided $1,499,988 per year over 5 years, for a total of $7,499,940. Multiple Power to Decide staff supported Innovation Next, including a Project Director with partial time on the project and at least one full-time Project Manager over all 5 years. Additional Power to Decide staff also had partial time on the project as they served in coaching roles. Each in-person workshop required between 7 and 10 staff, including the five staff serving as coaches to ensure individualized team support.

### Data Collection for Evaluation

To help identify needed adjustments to the implementation model, members of innovation teams completed an online survey administered via Alchemer (formerly SurveyGizmo) when they entered the Innovation Next process, before and after each workshop, and after the final pitches. These surveys collected data on knowledge about HCD, confidence using HCD strategies, and understanding of end user needs. We also asked them about their experiences participating in Innovation Next, including the utility of workshops, coaching calls, and webinars. Individual, in-depth interviews were conducted with at least one representative from each innovation team following the pitch sessions, and we periodically followed-up with teams during the remaining years of the project. These interviews addressed satisfaction with the Innovation Next process and the TA Power to Decide provided, as well as promising practices and lessons learned to inform future efforts. Descriptive and thematic analysis of the quantitative and qualitative data, respectively, along with observations and reflections from Power to Decide staff, were used to identify key implementation findings reported below.

## Implementation Findings

We identified eight key implementation findings that collectively reflect programmatic successes and challenges as well as barriers and facilitators. These finding include: (1) the process supported developing empathy for the end user; (2) responsive coaching was critical to success; (3) play and prototyping were important components of training; (4) assumptions were challenged and pivoting was necessary; (5) the process was not as easy as anticipated; (6) competition may have limited collaboration; (7) sustainability and scalability remain challenging; and (8) innovators changed how they approached their work beyond Innovation Next. Each of these is described in detail below.

### The Process Supported Developing Empathy for the End User

Developing empathy for the intended end users and using their feedback to define the problem and identify a solution are critical for HCD (Brown, 2008). Innovators gathered information from end users throughout the Innovation Next process. Frequency and methods for gathering feedback from end users varied depending on the team and the design challenge being addressed. At a minimum, these methods involved individual and group interviews, but also included HCD-specific strategies like journey mapping and analogous experiences. These strategies helped teams build empathy for end users. Journey maps, for instance, helped teams identify pain points, or points in a journey where end users experienced frustration or confusion, that could potentially be resolved through design features, while analogous experiences allowed teams to experience first-hand the emotions that their end users experienced by participating in an activity in a parallel context.

Innovators were asked at three time points how confident they felt seeking feedback from end users in order to define the solution—before the first workshop, after the workshop, and after the final pitch. Generally, team members became increasingly confident in their ability to engage end users in order to better understand end user experiences and perspectives related to their design challenge, with an almost 1.5-point mean increase in their confidence recruiting and conducting interviews with end users (on a five-point scale). As one participant said, “working with teens on a day-to-day basis, you think you know teens, but the design thinking process changes things. We do everything very different based on those [end user] interviews.” Four of the teams from Cohort 1 established advisory boards that included youth who were representative of their specific end user audiences or had young people formally working on the project on an ongoing basis. These findings suggest that the workshops and TA provided through Innovation Next reinforced the importance of engaging with and gaining empathy for end users.

### Responsive Coaching Was Critical to Success

Power to Decide integrated coaching throughout Innovation Next—teams engaged with a dedicated coach (who was a Power to Decide staff member trained in the HCD process) in-person during the workshops and by phone in between workshops. Some teams elected to meet with their coach monthly, particularly during the early part of the process. Rather than following a scripted format or predetermined set of activities, Innovation Next coaches were responsive to the specific needs of the teams. The coaches appropriately challenged teams to help them move forward, provided support when they were feeling discouraged, and shared their expertise when teams encountered barriers to the HCD process. Teams overwhelmingly spoke of how their coaches demonstrated empathy and adapted the content of TA calls to what they needed to make progress. Several teams indicated that they would not have been successful at developing or delivering the final product without the support of their coach. As one participant said, “Aside from technical skills that [our coach] has, she also was invested in us and bolstered us…Support is not a nice-to-have—it was critical to help us keep going.”

### Play and Prototyping Were Important Components of Training

Innovation Next promoted play as a key aspect of the workshops and included materials such as clay, slime, Legos, and pipe cleaners to stimulate creativity. Workshop facilitators aimed to establish a norm that no idea was a bad idea and encouraged teams to think outside-the-box by elevating ideas that were aspirational, extreme, or highly creative. Innovators also had the opportunity to test prototypes of unconventional ideas in order to build their capacity to develop and test prototypes of their own novel designs. The survey data indicated that teams universally found the workshops enlightening, supportive, and motivating. They valued the hands-on pedagogical approach, and several innovators specifically expressed appreciation for the opportunity to brainstorm freely and entertain big ideas that deviated from more traditional models of TPP. Although teams rarely moved forward with developing their initial ideas, they were an important starting point for a more resonant but still innovative approach. For example, one team proposed an experiential, museum-based experience where youth could learn about biology, safety, empathy, feelings, and other topics related to adolescent development and sexual health. While the concept of a museum was not ultimately pursued, the team used this idea to solicit end user feedback and then developed experiential learning opportunities that could be delivered in a digital or in-person environment. The prototyping exercises during the workshop also helped teams develop skills to facilitate development of their innovation. As one participant noted, “I had a mental block on how to prototype a service. The brainstorming tools really helped, and using existing resources to get a sense of what worked and what might be a different approach. We felt better prepared to develop prototypes for our own innovation.”

### Assumptions Were Challenged and Pivoting Was Necessary

Even among those who have been deeply involved in TPP in their community, Innovation Next challenged innovators’ assumptions about what young people need. All teams indicated that the HCD process was instrumental in developing their ideas, although not always in the ways they expected. For example, one team initially intended to focus on helping adolescents become more comfortable talking with their provider about contraception. However, after interviewing young people as an essential step in HCD, they realized that a more fruitful approach would be to help providers navigate adolescents’ discomfort:

“We went in looking for what it is that prevents you from having an uncomfortable conversation [about sex]. We didn’t find anything…it is just the nature of the beast. But learning about the constant concern of judgement was the biggest thing. How can we help providers create an environment that isn’t judgmental and all the verbal and non-verbal things that they do that conveys that judgement? Our very first interview told us so many things that her provider could have done differently.”

Another team initially thought that they would improve sex education in schools but found that their end users preferred a different source of information. A team member noted, “What was surprising for us was that teens just don’t want to hear about sex in school. And that they would want to learn about sex from hearing the stories from other teens. Text was boring but text messages were not.” This team revised their plan and instead developed an app where teens learn from peers in addition to experts. Teams’ ability to pivot was important for success. The strongest teams we observed allowed diverse new ideas to emerge and embraced a wide range of potential options before using guidance from end users to converge on a solution. In fact, the three teams with the highest scores in the Cohort 1 pitch session all reported a substantial shift in the development of their idea.

Innovation Next included a thorough prototyping phase, which allowed for more real-time feedback from end users. As a result, teams had plenty of opportunities to pivot as well as to ensure that they were addressing the right problem with the right solution. For example, many teams realized that they wanted to use a technology solution that was not the right fit for their audience. One team initially developed an app for teens to learn more about healthy relationships. However, in testing the prototype, they learned that teens were hesitant to download another app to their phones. Instead, they were interested in hearing more about healthy relationships through social media. As a result of this feedback, the team developed characters and storylines about healthy relationships for Instagram. A member of the team summarized this pivot:

“Our [initial] idea was very different [from the final product]—still cousins, but distant cousins. The goal never changed. We knew we wanted to reach a very specific population, but we shifted how we did it. When we started to talk to teens, they just weren’t going to use the solution we originally thought was best. Elements of that are still in there [the final product], but it is better.”

As another example, one team focused early prototyping on converting a camper van to a mobile clinic that could be available for groups of teens. However, the end users they interviewed reported that they would not want to interrupt time with their friends to visit the clinic van. Accordingly, the team pivoted to improve online appointment scheduling for traditional clinic visits, making it easier for teens to make appointments at times convenient for them. Other common reasons for pivoting included feasibility limitations and cost considerations. Innovators who were able to follow feedback from the end user during prototyping strengthened their end products.

### The Process Was Not as Easy as Anticipated

Some innovators described the HCD process as a roller coaster given the iterative nature of the work, which sometimes meant that progress stalled, or even regressed, before breakthroughs were made. One participant noted, “It has been a rollercoaster of emotions including complete overconfidence, success, and utter defeat.” Innovators’ confidence in their proposed solution decreased after the first workshop (which was around the time that they were first conducting interviews with end users) and then increased after the pitch. A participant summarized this evolution: “As messy as this process was, it only made us more confident in the end product.”

Some of the teams expressed that one of the biggest challenges with the HCD process was being open-minded to all feedback, rather than using engagement with end users to validate innovators’ initial proposed solution. Pivoting during the prototyping was also difficult for some teams. One participant noted, “We jumped quickly into the design thinking methodology, but some of the people in that room thought of design thinking as rules to apply versus thinking of them as tools to use and being willing to get messy with those tools…Set the stage for a lot of failure. Let them know that from the beginning.” Responding to this feedback, the Innovation Next application process was revised after Cohort 1 to require applicants to include two potential ideas in their application instead of just one. Doing so helped to establish expectations around flexibility early on and to select innovators who would be more open to pivoting.

### Competition May Have Limited Collaboration

As noted in the description of the implementation model, teams participating in Cohort 1 competed for funding to move from Stage 1 to Stage 2, and only half of participating teams moved forward. Teams from Cohort 1 had mixed feelings about the competitive process. While they appreciated that it motivated them to work hard and generate an amazing product idea, the competitive process may also have limited information-sharing and collaboration. Two teams reported that they wanted to share more across teams, but the competition discouraged them from doing so. As one participant said, “I wanted to hear more about what the other teams were doing—it felt like a missed opportunity not to share what we were learning…that felt risky because we were also in competition with them with the pitch.” For example, one participant suggested that the online discussion forum established to facilitate information-sharing may have been underutilized because teams were hesitant to divulge information that would give others a competitive advantage. This feedback informed the decision to revise the implementation model for Cohorts 2 and 3 and remove the competitive element.

### Sustainability and Scalability Remain Challenging

At one point, federal funding for Innovation Next was uncertain, and several teams from Cohort 2 reported that they lost momentum during this time. In some cases, team members were re-assigned to other projects, and even if they were able to return to the effort once funding was secured, additional time was needed to re-engage and apply the HCD processes during the period leading up to the pitch. More generally, almost every team experienced personnel changes. Team personnel changes frequently occurred in response to changing needs in skills as the teams progressed through the HCD process, in which case turnover was less of a barrier to sustainability. Other personnel departures were more challenging for teams. As one participant stated, “When we lost [NAME REDACTED], it was hard. He was really core to the work and we had to change our approach to future growth without him.”

Sustainability beyond Innovation Next is critical to scalability, though it was not expected to occur within the scope and timeline of the initiative. Once funding from Innovation Next ended, teams struggled to identify additional financial support, as there is limited funding available for interventions that do not yet have rigorous evidence of impact. Teams have explored a variety of options for sustaining their work and moving to scale, which requires significant investment in infrastructure and marketing. Potential strategies have included seeking additional accelerator-based funding, exploring investment and grant opportunities, and selling the product direct-to-consumers, using proceeds to support implementation in schools and nonprofits at no cost. One participant described sustainability and scalability as “the most challenging task so far,” noting “we have talked to VCs [venture capitalists] and app marketing experts, but I’m not sure we have figured out the special sauce that allows us to monetize it and keep trust with our users.” Despite the wide variety of approaches being pursued, funding for many teams and products remains uncertain moving forward.

### Innovators Changed How They Approached Their Work Beyond Innovation Next

Universally, teams expressed that participating in Innovation Next changed how they approach their work more broadly. Innovators indicated strong dedication to the HCD concepts they learned in the process, especially the need for greater empathy regarding the challenges young people face and the need to question long-standing approaches and assumptions, think creatively about problems, and obtain feedback as solutions are pursued. One participant said, “This process has been life changing in my work—the radical empathy perspective has influenced everything we do! The transferability of the work we’ve done, and lessons learned has been helpful in all our work.” Specifically, the team reported engaging youth in other activities, such as website redesign. They also added young people to a formal advisory board for the organization. Another participant said, “Innovation Next has definitely—not even changed—but has defined how we do things now at my organization. It gave us a structure for how we do our work. Teens are now at the center of the work, and we’ve transferred this idea to everything we do—our board meetings, our retreats, our way of life here.”

## Discussion

Implementation of Innovation Next from 2015 to 2021 catalyzed technology-based innovation in the field of TPP. Multiple innovations developed as part of the initiative are now on the market, including: a scheduling system that facilitates easy and confidential booking of clinical appointments (BC4U, 2022); a mobile application that provides authentic stories and high quality online resources about topics such as puberty and healthy relationships (MyHealthEd, 2022); a mobile application that offers a forum for asking questions about sexual and reproductive health and receiving tailored responses from experts (OkaySo, 2022); and a digital toolkit for pharmacists to increase youth-friendliness and support access to emergency contraception (Miss Morning After, 2022). While an important next step is to rigorously evaluate effectiveness, these interventions have expanded available TPP programming in a meaningful way, providing technology-based options specifically designed to meet the needs of young people today.

Although Innovation Next was developed in response to a specific federal funding opportunity, the implementation model and findings can inform future efforts to develop innovative, technology-based TPP programs using HCD. We recommend that incubator programs are designed to facilitate collaboration between participants rather than competition and that structured training and coaching provided by experts in HCD are included as foundational components. The workshops and responsive coaching central to Innovation Next helped build innovators’ knowledge and skills regarding the HCD process and importantly, facilitated application specific to TPP. In general, we found that more frequent coaching and touchpoints were beneficial in supporting teams as they built their capacity to implement HCD principles. Although teams demonstrated openness to a variety of ideas, an important attitude for innovation (Basadur et al., 2000), the process was not seamless, further underscoring the value of a structured implementation model to deliver training and coaching. Formal training, tools, and positive support and feedback have also been noted as facilitators in other HCD initiatives (Liedtka et al., 2020; Zuber & Moody, 2018). One of the central outcomes of the training and TA was team implementation of end user engagement activities to support the development of radical empathy for young people. Such engagement with end users should be standard practice of any innovation implementation model (Brown, 2008) and occur not only in the initial development of a concept or “new” idea but during all phases of intervention development. Agencies funding innovative products and ideas would benefit from allowing time and resources to engage end users throughout the development process as well as encouraging grantees to pivot from initial plans based on end user feedback.

Barriers to implementation we identified included challenges shifting mindsets from traditional program development methods, competition limiting collaboration, and issues with sustainability and scalability, largely due to funding constraints and staff turnover. Uneasiness with the HCD process, which does not rely on a predetermined outcome, has been previously documented (Ostrach, 2020; Vechakul et al., 2015). Many of the cohorts included innovators with a long history of working on adolescent sexual and reproductive health and developing interventions using more traditional methods. It has been recognized that expertise in a field can, in some cases, limit the development of novel ideas (Dan, 2010; Moreau et al., 2001). Expertise can still be valuable, but establishing the need for flexibility early on, including setting the expectation that pivoting will likely be necessary, may help professionals apply HCD principles more easily. The revised approach used with Cohorts 2 and 3 is one solution to the barrier associated with competition—it is possible for teams to develop and deliver a pitch without truly competing in order to facilitate information-sharing during this stage. As for sustainability and scalability, the TPP field may need to consider additional mechanisms to support projects beyond the initial development of an MVP but before there is rigorous evidence of effectiveness. A gap in available support at the federal level to move from innovation to evidence has already been noted (Diana & Bennett, 2015).

Beyond resulting in the development of innovative TPP interventions, a notable success of Innovation Next is that it led to meaningful change in how innovators approached their work beyond this specific initiative, with more explicit attention to youth engagement. Such impacts at the organizational level may help sustain innovation in the long term. Changing mindsets and organizational culture has been described elsewhere as an outcome of HCD (Liedtka et al., 2020), but Innovation Next is particularly unique given the scale at which HCD was implemented. With 60 individuals trained across more than 40 organizations, the model has infused HCD into the TPP field.

As innovation is pursued to support TPP goals, technology-based approaches are clearly important, and HCD offers a practical framework for catalyzing new technologies that meet the needs of young people. Going forward, HCD should be supported as part of public health and clinical efforts to promote adolescent sexual and reproductive health. Innovation Next can serve as a model for implementation with clear potential for impact—transforming innovation teams, organizations, the broader field, and ultimately, the lives of adolescents.

## References

[CR1] Abookire S, Plover C, Frasso R, Ku B (2020). Health design thinking: An innovative approach in public health to defining problems and finding solutions. Frontiers in Public Health.

[CR2] Anderson, M. & Jiang, J. (2018). *Teens, social media & technology.* Pew Research Center. Retrieved June 23, 2022, from: https://www.pewresearch.org/internet/2018/05/31/teens-technology-appendix-a-detailed-tables/

[CR3] Antonishak J, Kaye K, Swiader L (2015). Impact of an online birth control support network on unintended pregnancy. Social Marketing Quarterly.

[CR4] Basadur MM, Runco MA, Vega LA (2000). Understanding how creative thinking skills, attitudes and behaviors work together: A causal process model. Journal of Creative Behavior.

[CR5] Bazzano, A.N., Martin, J., Hicks, E., Faughnan, M., & Murphy, L. (2017). Human-centereddesign in global health: A scoping review of applications and contexts. *PloS One,* 12. 10.1371/journal.pone.0186744 10.1371/journal.pone.0186744 PMC5665524 29091935

[CR6] BC4U. (2022). About us. Retrieved June 27, 2022, from https://bc4u.org/about-us/

[CR7] Brown, T. (2008). Design thinking. *Harvard Business Review, 86,* 85–92*.* https://hbr.org/2008/06/design-thinking 18605031

[CR8] Brown, T., & Wyatt, J. (2010). Design thinking for social intervention. *Stanford Social Innovation Review, 8*, 30–35. https://ssir.org/articles/entry/design_thinking_for_social_innovation

[CR9] Dan E (2010). Reconsidering the trade-off between expertise and flexibility: A cognitive entrenchment perspective. Academy of Management Review.

[CR10] Diana, A., & Bennett N. (2015). Federal mechanisms to support intervention dissemination. *New Directions for Child and Adolescent Development, 149,* 69–79.10.1002/cad.20114 10.1002/cad.20114 26375192

[CR11] Liedtka, J., Sheikh, A., Gilmer, C., Kupetz, M., & Wilcox L. (2020). The use of design thinking in the U.S. federal government. *Public Performance & Management Review, 43*, 157-159. 10.1080/15309576.2019.1657916

[CR12] Lubana, P. (2021). *Practicing radical empathy in product design*. Retrieved November 15, 2022, from https://uxdesign.cc/a-case-for-radical-empathy-in-product-design-4afe82007b7f

[CR13] Miss Morning After. (2022). About us. Retrieved June 27, 2022, from https://www.missmorningafter.com/about-us

[CR14] Moreau, C.P., Lehmann, D.R., & Markham, A.B. (2001). Entrenched knowledge structures and consumer response to new products. *Journal of Marketing Research, 38,* 14–29. https://www.jstor.org/stable/1558568

[CR15] MyHealthEd. (2022). Inc. Real Talk. Retrieved June 27, 2022, from https://bc4u.org/about-us/

[CR16] Nandan M, Jaskyte K, Mandayam G (2020). Human centered design as a new approach to creative problem solving: Its usefulness and applicability for social work practice. Human Service Organizations: Management, Leadership & Governance.

[CR17] Office of Population Affairs. (2022). About TPP. Retrieved June 23, 2022, from https://opa.hhs.gov/grant-programs/teen-pregnancy-prevention-program-tpp/about-tpp

[CR18] OkaySo. (2022). About us. Retrieved June 27, 2022, from https://okayso.org/about-us

[CR19] Osterman MJK, Hamilton BE, Martin JA, Driscoll AK, Valenzuela CP (2022). Births: Final data for 2020. National Vital Statistics Reports.

[CR20] Ostrach, B. (2020). Human-centered design for a women's health screening tool: Participant experiences. *Southern Medical Journal*, *113*, 469–474. 10.14423/SMJ.0000000000001157 10.14423/SMJ.0000000000001157 33005959

[CR21] Razzouk, R., & Shute, V. (2012). What is design thinking and why is it important? *Review of Educational Research, 82,* 330–348. https://doi.org.proxy.library.emory.edu/10.3102/0034654312457429

[CR22] Vechakul J, Shrimali BP, Sandhu JS (2015). Human-centered design as an approach for place-based innovation in public health: A case study from Oakland, California. Maternal and Child Health Journal.

[CR23] Widman L, Nesi J, Kamke K, Choukas-Bradley S, Stewart JL (2018). Technology based interventions to reduce sexually transmitted infections and unintended pregnancyamong youth. Journal of Adolescent Health.

[CR24] Zuber CD, Moody L (2018). Creativity and innovation in health care: Tapping into organizational enablers through human-centered design. Nursing Administration Quarterly.

